# Patient Characteristics and Clinical Outcomes During the 2020–2021 COVID-19 Wave: An Observational Study at a Tertiary Hospital in Saudi Arabia

**DOI:** 10.7759/cureus.71119

**Published:** 2024-10-09

**Authors:** Omar B Ahmed, Atif Asghar, Majid Bamaga, Ibrahim H Abd El-Rahim, Bassam Mashat, Asim Khogeer, Hamza Assaggaf

**Affiliations:** 1 Environmental and Health Research, Umm Al-Qura University, Makkah, SAU; 2 Institutional Review Board, General Directorate of Health Affairs Makkah Region, Ministry of Health, Makkah, SAU; 3 Research Office, General Directorate of Health Affairs Makkah Region, Ministry of Health, Makkah, SAU; 4 Laboratory Medicine/Public Health, Umm Al-Qura University, Makkah, SAU

**Keywords:** covid-19, demographics, laboratory findings, mortality, severity

## Abstract

Background and objective

The continued prevalence and threat of coronavirus disease 2019 (COVID-19) have been reported, and evidence suggests that several people still get infected with the virus. Gaining a thorough understanding of the patient demographic factors and laboratory findings could contribute to assessing the severity, mortality, and progression of COVID-19. In light of this, the current study aimed to evaluate the demographic characteristics, laboratory findings, and outcomes of confirmed COVID-19 patients at a tertiary hospital in the Kingdom of Saudi Arabia (KSA).

Methodology

We collected data spanning the period 2020-2021 from the electronic health records of Al-Noor Specialized Hospital, Ma including demographics (age, gender, and nationality), severity (i.e., ICU admission), length of hospital stay, mortality, and laboratory parameters.

Results

We observed an overall mortality rate of 10.2% (338 of 3,307 patients). The mortality rate was significantly higher in males (n=210; 62.1%) and patients aged more than 70 years (n=91; 26.9%). Patients with blood group O comprised 131 (29%) of the 338 non-survivors, followed by those with A (n=85; 25.1%) and B groups (n=79; 23.4%). The mortality rate among ICU patients was 63.3% (n=214). Furthermore, the following laboratory findings showed abnormal mean values in terms of severity and mortality in COVID-19 patients: hemoglobin (HB) concentration, white blood cell (WBC) count, lymphocyte count (LC), C-reactive protein (CRP), creatinine (CREA), and uric acid (UA) levels.

Conclusions

Old age, male gender, and certain laboratory findings have a critical role in the severity and mortality risk in COVID-19 patients. There was no significant association between blood type and the severity and mortality of COVID-19. Continuous monitoring based on these findings may be essential to managing COVID-19 patients.

## Introduction

As of May 2024, coronavirus disease 2019 (COVID-19) continues to impact the global population, although the situation varies significantly by region. According to the World Health Organization (WHO), the rate of new COVID-19 cases increased by 4% from mid-December 2023 to early January 2024, with over 1.1 million new cases reported worldwide [1]. As the virus spreads and transforms, variations in its genetic makeup give rise to different COVID-19 strains. These mutations may change the severity of the disease, increase its transmissibility, and affect the efficacy of vaccines [2,3]. Examples of such mutations include the Alpha (B.1.1.7) variant, which was first identified in the United Kingdom in September 2020, and the Beta (B.1.351) variant, which was first identified in South Africa in May 2020 [2]. Other variants with high transmissibility have also been identified, such as the Gamma (P.1), Delta (B.1.617.2), and Omicron (B.1.1.529) variants; while these caused less severe disease, they also dulled the efficacy of the COVID-19 vaccine [4].

The severity, mortality, demographics, and laboratory parameters of COVID-19 outbreaks can vary significantly in different regions due to socioeconomic conditions and the effectiveness of public health measures. The common symptoms of COVID-19 include fever, cough, and shortness of breath, while some of the other observed complications include pneumonia, acute respiratory distress syndrome, septic shock, and acute kidney injury. Older age, male gender, high BMI, and the presence of multiple comorbidities are associated with higher rates of ICU admission and mortality [5]. Blood type has a minor overall impact on COVID-19 outcomes, although blood group A is linked to higher severity and mortality due to severe consequences such as thrombosis [6], while blood group O may provide some protection against COVID-19 and is associated with a lower risk of the disease [7].

Routine laboratory monitoring is important in the management, epidemiologic surveillance, and therapeutic monitoring of COVID-19 patients [8]. White blood cell (WBC) counts and concentration of hemoglobin (HB) may be significant factors in predicting the clinical results and mortality of COVID-19 patients [9]. Abnormal WBC, including lymphopenia and an increased neutrophil-to-lymphocyte ratio (NLR), are significant indicators of mortality in COVID-19. Another key biomarker in predicting the severity and mortality of COVID-19 patients is the C-reactive protein (CRP). The liver produces CRP, an acute-phase protein, in reaction to inflammation, especially when cytokines such as interleukin-6 (IL-6) are present. COVID-19 patients, especially severe cases, often have elevated CRP levels, which are highly correlated with worse outcomes such as hospitalization or ICU admission and increased mortality [10]. The prognosis of COVID-19 patients is also significantly influenced by renal function, which is assessed by serum creatinine (CREA) level measurement. In addition to the lungs, the kidneys are among the organs that the virus can infect. A major side effect of COVID-19, especially in patients who are critically ill, is acute kidney damage, which has been linked to worse outcomes and higher fatality rates [11].

The city of Makkah in the Kingdom of Saudi Arabia (KSA) is one of the most crowded cities in the world, especially during the Islamic holy months of Ramadan and Dhu al-Hijjah, when millions of Muslims travel to perform Umrah and Hajj. The large gatherings of people during these times lead to an increased risk of COVID-19 transmission [12]. The current study aimed to evaluate patient characteristics and clinical outcomes during the 2020-2021 COVID-19 wave at a tertiary hospital in Makkah.

## Materials and methods

Study design

Type of Study

A retrospective observational cross-sectional study was conducted to assess the characteristics, laboratory findings, severity, and clinical outcomes of COVID-19 patients during the 2020-2021 wave at the Al-Noor Specialized Hospital in Makkah, KSA

Study Period and Setting

The study examined patients admitted between March 2020 and December 2021, which constituted the peak of the COVID-19 wave in that region. The study was conducted at a tertiary hospital that provides advanced medical care, including specialized COVID-19 units, ICUs, and access to mechanical ventilation.

Data collection

Data Sources and Variables

Data were collected from the electronic health records (EHR) of Al-Noor Specialized Hospital. The data included demographics (age, gender, and nationality), severity, ICU admission, length of hospital stay, and mortality. The laboratory parameters measured after the onset of COVID-19 included blood group (ABO), HB concentration, WBC count, and lymphocyte count (LC) as well as CRP, CREA, and uric acid (UA) levels. The variables included in our study were demographic information (age, sex, and nationality), laboratory findings (ABO blood type, HB concentration, WBC count, LC, CRP, CREA, and UA), ICU admission data (need for intensive care and duration of ICU stay), and patient outcomes (length of hospital stay, discharge status, survival, and mortality).

Study population

Inclusion Criteria

Patients living in the city of Makkah who were diagnosed with COVID-19 through a real-time polymerase chain reaction (RT-PCR) test and admitted to Al-Noor Specialized Hospital between March 2020 and December 2021 were deemed eligible for inclusion.

Exclusion Criteria

Patients whose RT-PCR test was negative, who had incomplete medical records, or who were transferred to other facilities before outcome data could be obtained were excluded

Ethical considerations

The ethical approval was obtained from of Institutional Review Board (IRB) of the Directorate of Health Affairs (DHA) in Makkah Region (IRB no. H-02-K-076-0920-372, dated 04.10.2020). Ethical approval was obtained before data collection. The study adhered to the principles of the Declaration of Helsinki. Informed consent was not applicable as the study relied on electronic health records and not direct patient contact.

Data analysis

Firstly, the means and standard deviations (SD) for continuous variables (e.g., age) were calculated. Next, the frequencies and percentages for categorical variables (e.g., gender, nationality, and age) were determined. Finally, student t-tests and chi-square tests for categorical variables were performed to compare differences in mortality based on gender, age group, nationality, laboratory findings, and blood type testing.

## Results

Table 1 shows the demographic characteristics of the 3,307 patients included in this study. The mean age of the patients was 50.3 ±15.54 years; 2,218 (67.1%) were male, while 1089 (32.9%) were female. The number of patients in the age group of 51-60 was 777 (23.5%). Of note, 1,199 patients (36.3%) were Saudi nationals, while 523 (15.8%) were from Myanmar, and 324 (9.8%) were from Bangladesh. The total number of patients admitted to the ICU was 822 (24.9%). Out of 1,448 whose blood group was known, the highest incident rate was among patients with type O blood (574; 39.6%), followed by patients with type A blood (450; 31.1%) and type B blood 350 (24.2%). The mean length of stay (LOS) in the ICU was 12.5 ±8.20 days.

**Table 1:** General demographic characteristics and blood type among the cohort ICU: intensive cure unit; LOS: length of stay; SD: standard deviation

Characteristics	Results	Total number
Demographics		
Age, mean +SD	50.3 +15.54	3,307
Age groups, years, n (%)		
<20	49 (1.5)	3,307
21-30	274 (8.3)	
31-40	609 (18.4)	
41-50	734 (22.2)	
51-60	777 (23.5)	
61-70	505 (15.3)	
More than 70	359 (10.9)	
Gender, n (%)		
Male	2,218 (67.1)	3,307
Female	1,089 (32.9)	
Nationality		
Saudi	1,199 (36.3)	3,307
Myanmar	523 (15.8)	
Bangladesh	324 (9.8)	
Egypt	266 (8)	
Pakistan	262 (7.9)	
Yemen	215 (6.5)	
India	147 (4.4)	
Afghanistan	51 (1.6)	
Syria	42 (1.3)	
Indonesia	37 (1.1)	
Sudan	33 (1.0)	
Nigeria	32 (1.0)	
Other	176 (5.3)	
ICU		
Admitted to ICU, n (%)	822 (24.9)	3,307
Mortality, n (%)	338 (10.2)	
ICU LOS, days, mean ±SD	12.5 ±8.20	
Blood group, n (%)		
A	450 (31.1)	1,448
AB	74 (5.1)	
B	350 (24.2)	
O	574 (39.6)	

The overall mortality rate (non-survivors) was 10.2% (n=338). The demographic characteristics (gender, age, nationality, and ICU admission) and mortality of the survivors and non-survivors were compared using chi-square tests (Table 2). In general, there were significant differences (p<0.05) in terms of gender, nationality, severity (ICU admission based on severity), and blood group. Of the 338 non-survivors, 210 (62.1%) were male and 128 (37.9%) were female (p<0.05), while 26.9% (91) were aged more than 70 years. Furthermore, the mortality rate was higher among ICU patients (214; 63.3%) than non-ICU patients (124; 38.8%). As for blood type, the highest mortality rate was among the O blood group patients (n=131, 29%), followed by the A blood group patients (n=85, 25.1%) and B blood group patients (n=79, 23.4%).

**Table 2:** Comparisons of gender, age, ICU admission, and blood type between survivors and non-survivors ICU: intensive cure unit

Variables	Non-survivors, n (%)	Survivors, n (%)	P-value
Gender			0.036
Male	210 (62.1%)	2,008 (67.6%)	
female	128 (37.9%)	961 (32.4%)	
Total	338 (100%)	2,969 (100%)	
Age group (years)			0.000
≤20	2 (0.6%)	47 (1.6%)	
21-30	14 (4.1%)	260 (8.8%)	
31-40	28 (8.3%)	581 (19.6%)	
41-50	53 (15.7%)	681 (22.9%)	
51-60	69 (20.4%)	708 (23.8%)	
61-70	81 (24.0%)	424 (14.3%)	
>70	91 (26.9%)	268 (9.0%)	
Total	338 (100%)	2,969 (100%)	
Severity (ICU)			0.000
ICU admission	124 (36.7%)	698 (23.5%)	
Non-ICU admission	214 (63.3%)	2,271 (76.5%)	
Total	338 (100%)	2,969 (100%)	
Blood group			0.017
A	85 (25.1%)	365 (32.9%)	
AB	43 (12.7%)	31 (2.8%)	
B	79 (23.4%)	271 (24.4%)	
O	131 (38.8%)	443 (39.9%)	
Total	338 (100%)	1,100 (100%)	

**Figure 1:**
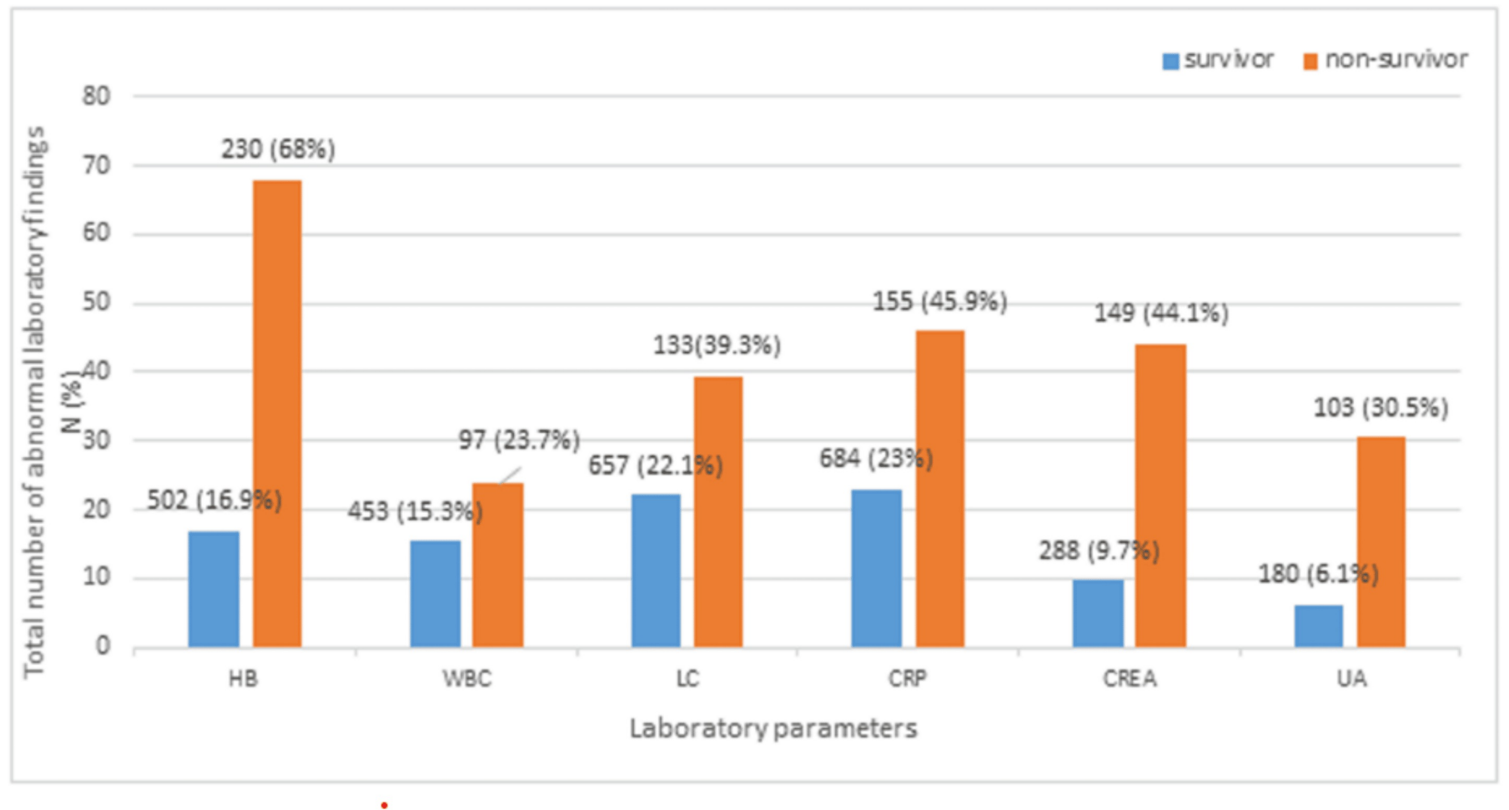
Comparison of abnormal laboratory findings between survivors and non-survivors in the cohort CREA: serum creatinine; CRP: C-reactive protein; HB: hemoglobin concentration; LC: lymphocyte count; UA: uric acid; WBC: white blood cells

As shown in Table 3, laboratory findings (CRP, WBC count, LC, HB concentration, CREA levels, and UA levels) were categorized as normal and abnormal and compared to the mortality variable (survivors and non-survivors). The total number of abnormal results in terms of various parameters were as follows - CRP: 155 (45.9%), WBC count: 97 (28.7%), LC: 133 (39.4%), HB concentration: 230 (68%), CREA level: 149 (44.1%), and UA level: 103 (30.5%). In general, all laboratory findings (CRP, WBC count, LC, HB concentration, CREA levels, and UA levels) revealed abnormal mean values and significant differences (p<0.05) among COVID-19 patients, particularly non-survivors (Figure 1).

**Table 3:** Initial laboratory findings among the cohort CREA: serum creatinine; CRP: C-reactive protein; HB: hemoglobin concentration; LC: lymphocyte count; SD: standard deviation; UA: uric acid; WBC: white blood cells

Laboratory tests	Initial results, mean ±SD	Normal range	Abnormal results (survivors, n=2969), n (%)	Abnormal results (non-survivors, n=338), n (%)	P-value
HB, g/dL	12.8 ±2.4	12–17.5	502 (16.9%)	230 (68%)	0.00
WBC, × 10^3^/μL	8.3 ±5.5	4-11	453 (15.3%)	97 (23.7%)	0.00
LC, × 10^3^/μL	1.7 ±1.3	1.1–3.2	657 (22.1%)	133 (39.3%)	0.00
CRP, mg/L	7.3 ±6	<8	684 (23%)	155 (45.9%)	0.00
Crea, umol/L	153.1 ±219.4	44-133	288 (9.7%)	149 (44.1%)	0.00
UA, umol/L	340 ±173.3	<300	180 (6.1%)	103 (30.5%)	0.00

## Discussion

COVID-19 has led to significant morbidity and mortality worldwide. However, very few studies have addressed the diagnostic importance of abnormal laboratory findings in COVID-19 patients. Understanding laboratory findings in COVID-19 patients can help aid in disease prognosis and reaching favorable patient outcomes. The present study focused on how demographic characteristics and different laboratory parameters correlated with mortality in confirmed COVID-19 patients at a tertiary hospital in KSA. Our findings showed that there was a higher prevalence of COVID-19 among male patients (n=2,218, 67.1%), those in the age group of 51-60 years (n=777, 23.5%), Saudi nationals (n=1,199, 36.3%), and patients with O blood group (n=574, 39.6%). The total number of patients admitted to the ICU was 822 (24.9%), and the mean LOS in the ICU was 12.5 ±8.20 days.

We observed significant differences (p<0.05) in terms of gender, nationality, severity (ICU admission), and blood group. Thus, demographic factors appear to have a potential association with the severity and mortality of COVID-19. Several studies have demonstrated that elderly people are more severely affected than younger people, and males experience higher rates of severity and mortality compared to females [13,14]. In contrast, a retrospective observational study conducted at one hospital (Nord Franche-Comté) involving 54 patients with confirmed COVID-19 found that 67% of COVID-19 patients were female [15]. In another study, the mean age of COVID-19 patients was 66 years, and the mortality rate was 27%, with most fatal cases (90%) reported in patients aged 65 years or older [16]. However, one study reported that the median age of COVID-19 patients was 36 years; 54.3% of the patients were male, and 4.7% of them required ICU treatment [17].

In another study undertaken in Saudi Arabia, males constituted 71.7% of COVID-19 patients; there were more non-Saudi than Saudi patients, and mortality was most frequently reported in patients aged 50−59 years [18]. Another study reported a fatality rate of 15% [19]. The mean LOS in the ICU was found to vary depending on the severity of the illness and patients’ health conditions. For instance, a shorter ICU stay (ranging from a few days to around 10 days) was often found in mild to moderate cases, but those with severe cases of COVID-19 often had a longer ICU stay (up to 21 days) [20,21]. The results of another study demonstrated a direct effect of blood group A on viral infection, suggesting that individuals with type A blood may exhibit an increased susceptibility to COVID-19 as a result of direct engagement of the blood group A antigen [22]. In line with the results of the current study, a cohort study showed that 48.8% of COVID-19 patients had type O blood [23].

In the present study, the mortality rate was high, particularly in males, patients aged more than 70 years, ICU patients, and those with blood group O. Globally, many studies have shown fluctuating patterns in terms of the association between mortality rates and age groups, gender, education level, race/ethnicity, comorbidities, and regions [24-26]. Serratrice et al. [24], in a study on long-term mortality among very old COVID-19 survivors, found no excess mortality over an 18-month period. In contrast, Nguyen et al. [25], in a large-scale analysis of COVID-19 adult patients, reported that males had a higher rate of respiratory intubation, longer hospital stays, and a higher mortality rate vs. females, even when accounting for age, race/ethnicity, insurance status, and comorbidities. Similarly, Pothisiri et al. [26] examined gender differences in relation to excess mortality during the COVID-19 pandemic in Thailand. They reported that gender inequality in mortality was evident across age groups and regions. They emphasized the need for greater focus on gender disparities in mortality and called for targeted interventions to address these issues. A study conducted in Saudi Arabia has reported that the mortality rate was higher among males and those aged over 45 years [27]. 

Regarding the blood group, one study found that B+ blood group patients comprised 54.6% of COVID-19 patients and had a 100% mortality rate, while O blood group patients had a mortality rate of 92.9%. This suggests the importance of genetic considerations in understanding disease outcomes [28]. In contrast, one study reported that the highest mortality rate was found among blood group A patients (13.9%), followed by group B (9.5%), and group AB (10.2%) patients; there was no significant association between blood groups and severity and mortality of the COVID-19 disease [23]. In light of these varying findings, the current study suggests that there is no association between blood type and the severity and mortality of COVID-19. Although certain blood types, such as type O, may be linked to a higher risk of COVID-19 severity and mortality, the overall impact of blood type on outcomes is still small. Other factors, such as age, comorbidities, and overall health, play a much more significant role in determining patient outcomes in COVID-19. As for other laboratory findings, this study showed more frequent abnormal laboratory findings in terms of CRP (45.9%), WBC count (28.7%), LC (39.4%), HB (68%), CREA (44.1%), and UA levels (30.5%) among non-surviving patients vs. survivors.

WBC and HB concentration may be significant factors in predicting the clinical results and mortality in COVID-19 patients. The association between HB levels and COVID-19 mortality has indicated that patients with abnormal HB levels may have worse outcomes [9,29]. Similarly, the laboratory results in the study by Bassetti et al. [30] showed leukopenia in 25% of COVID-19 patients. Another study concluded that a higher CRP level can be an indicator of disease progression [13]. Furthermore, Bastug et al. [11] found abnormal creatinine levels to be a factor linked to increased severity of COVID-19. A study from Oman reported that high CRP levels, in addition to laboratory findings related to CREA and HB, could be significantly correlated with mortality rate [29]. Hence, laboratory parameters play a critical role in assessing the severity and mortality risk in COVID-19 patients. In general, all laboratory findings revealed abnormal means and a significant difference (p<0.05) among COVID-19 patients, particularly non-survivors.

This study has several limitations, including potential missing or incomplete data, the relatively small sample size, and its retrospective design (which limits the ability to establish temporality). Its observational design and the fact that the setting was confined to a single tertiary hospital may also be regarded as limitations. However, despite these limitations, the study provides a detailed exploration of patient data and outcomes, thereby offering important insights into the clinical course of COVID-19 and factors affecting patient prognosis.

## Conclusions

This study delved into the association of demographic factors, severity, and laboratory findings with mortality outcomes in COVID-19 patients. The findings showed that older age and male gender are associated with increased severity and higher mortality. Significant differences in gender, nationality, ICU admission, and blood group were observed between survivors and non-survivors. Abnormal laboratory findings, particularly in CRP, WBC count, LC, HB concentration, CREA, and UA levels, were more prevalent among non-survivors. These findings highlight the importance of demographic and laboratory markers in assessing the severity and outcomes of COVID-19 in hospitalized patients. Therefore, continuous monitoring based on these laboratory findings may be essential to manage COVID-19 patients.
